# Prevent, Promote, Promulgate^®^: A Realist Synthesis of Oral Health Outreach from Optics to Outcomes, Across Children, Communities, and the Special Care Continuum

**DOI:** 10.3390/children13091212

**Published:** 2026-09-08

**Authors:** Vaibhav Kumar, Praveen Jodalli, Ziad D. Baghdadi, Ramya Shenoy, Ishan Maliye, Ridhima Gaunkar

**Affiliations:** 1 Department of Public Health Dentistry, Manipal College of Dental Sciences Mangalore, Manipal Academy of Higher Education, Manipal 576104, Karnataka, India; 2 Department of Public Health Dentistry, Dr. G. D. Pol Foundation Y.M.T. Dental College and Hospital, Kharghar, Navi Mumbai 410210, Maharashtra, India; 3 Kartavya Disha Global Foundation, Mumbai 401202, Maharashtra, India; 4 TopSmiles Pediatric Dentistry and Orthodontics, Winnipeg, MB R2M3A4, Canada; 5 Butterfly Dental Group, Winnipeg, MB R3Y1P5, Canada; 6 Suitradhaar Strategies, Mumbai 400706, Maharashtra, India; 7 Department of Public Health Dentistry, Goa Dental College and Hospital, Bambolim 403202, Goa, India

**Keywords:** realist review, RAMESES, children’s oral health, school dental screening, special care dentistry, dental public health, referral completion, competency-based education, health equity

## Abstract

**Highlights:**

**What are the main findings?**

• Children’s oral health outreach is evaluated by optics, the number of children screened and photographed, rather than by verified outcomes such as referral completion and sustained care.
• A realist synthesis yields eight Context–Mechanism–Outcome configurations, six enabling and two antagonistic, organised as a Constellation of Care navigated by competency-based education.

**What are the implications of the main findings?**

• The intervention is not the camp but the configuration of conditions that lets it convert into care, so programmes should be judged on which child got better, not on which child was seen.
• Competency-based education, in paediatric and special care dentistry, is the generative lever that reorients practice from optics to outcomes, and the eight configurations are propositions for prospective realist evaluation.

**Abstract:**

**Background/Objectives:** Dental caries is the most prevalent chronic disease of childhood, and untreated caries in the primary dentition affects an estimated 514 million children worldwide. Children are the principal target of oral health outreach through school dental screening, community camps, and charitable events for children with special healthcare needs, yet these programmes are evaluated largely by what is most visible: the number of children examined and the photographs disseminated. This measurement substitution dissociates activity from impact, so that children are screened but not treated, counted but not cared for. The objective was to determine what works, for whom, in what contexts, and through what mechanisms in converting children’s oral health screening contacts into sustained care, with primary applicability to low- and middle-income, community-outreach-oriented health systems and, in particular, to India and South Asia, while the configurational logic is offered as transferable rather than universal. **Methods:** A realist synthesis was conducted and reported in accordance with the RAMESES publication standards. An initial programme theory was developed and refined through purposive iterative searching, appraisal of relevance and rigour, and the extraction of Context, Mechanism, and Outcome (CMO) configurations across the diverse literature. Searching ran from initiation to theoretical saturation; complete strategies, dates, and a document-selection flow diagram are provided. **Results:** Eight configurations were derived, six enabling and two antagonistic, organised around three thematic constellations, Prevent, Promote, and Promulgate, and navigated by a compass of competency-based education. Thirty-six documents were retained as the principal contributors to the final synthesis. The refined theory holds that the intervention is not the camp but the configuration of conditions that allows it to convert into care for the child. **Conclusions:** Children’s oral health outreach should be evaluated as a multi-level health-systems configuration rather than a discrete event; competency-based education is the generative lever that reorients practice from optics to outcomes. The eight configurations are theoretical propositions that require prospective empirical testing through realist evaluation before they are treated as confirmed causal claims.

## 1. Introduction

### 1.1. Rationale: Children, Camps, and a Realist Question

Two terms recur throughout this synthesis and are defined here at the outset to avoid ambiguity. By *optics* we mean the visible, readily counted markers of programme activity, such as the number of children screened, the number of camps held, and the photographs and social media reach generated, that are used, explicitly or implicitly, as evidence of programme value. By *outcomes* we mean the verified child-level results of that activity, such as referral completion, treatment received, sustained behaviour change, and recall attendance. The distinction is descriptive rather than pejorative: optics are necessary for accountability and mobilisation, and the concern of this synthesis is not that optics are recorded but that they are frequently *substituted* for outcomes that are never measured.

Dental caries is the most prevalent chronic disease of childhood, and untreated caries in the primary dentition affects an estimated 514 million children worldwide, a burden that falls disproportionately on low- and middle-income countries and on the poorest children within every country [1,2]. In much of the world, the dominant response has been outreach: school dental screening, anganwadi and community oral health camps, mobile dental units, and charitable events directed at vulnerable groups. Children are the principal beneficiaries of this activity, and school screening in particular is the archetypal children’s programme. The evaluation of that activity, however, has been troubled by a paradox. Where impact has been measured, it has usually been measured as activity, that is, the number of children screened and the number of camps held. Where genuine outcomes have been measured, namely referral completion, treatment received, and behavioural change, the picture is sobering. A rural Indian study documented substantial non-attendance after camp-based screening, driven by awareness, distance, affordability, and competing demands rather than refusal [3]. A 2022 Cochrane review concluded that the evidence that school dental screening improves attendance is of very low certainty and that improvement in oral health following screening remains unproven [4]. Dental care is, in parallel, the single most prevalent unmet health need reported for children in high-income settings, which shows that the gap between detection and care is not confined to resource-poor systems [5].

The realist approach, developed by Pawson and Tilley for complex social interventions [6,7], reframes the question from “*does it work?*” to “*what works, for whom, in what contexts, and through what mechanisms?*” Its unit of analysis is the Context, Mechanism, and Outcome configuration. This orientation suits children’s oral health outreach, which is a complex intervention mediated by families, schools, institutions, and the education of the workforce; which is rich in heterogeneous evidence that conventional hierarchies handle poorly; and whose central question is no longer whether outreach happens but under what conditions it converts into care for a child.

**Scope and applicability.** Although the problem of measurement substitution is observed across income settings, the evidence assembled here, and therefore the primary applicability of the framework, is concentrated in low- and middle-income, community-outreach-oriented health systems, with particular emphasis on India and South Asia, where school health programmes, anganwadi services, frontline health workers, and mobile dental units generate a high volume of screening contacts. High-income settings are drawn upon to show that the detection-to-care gap is not confined to resource-poor systems, but the configurations are proposed as transferable working theories to be re-specified against local context, not as claims of universal validity.

### 1.2. The Special Care Continuum and the Vulnerable-Child Optics Paradox

A specific and instructive population sits at the heart of this problem. Nearly one in five children has a special healthcare need, defined as a physical, developmental, behavioural, sensory, cognitive, or emotional condition that requires health intervention beyond what is generally required [8]. For these children, oral disease is more common, self-care is harder, and access to skilled dental services is more limited; dental care is the most frequently cited unmet health need for children with special healthcare needs, and the more severe the condition, the greater the unmet need [8,9,10]. Special care dentistry, the field concerned with people whose disability or medical or social circumstance affects oral health or its delivery, therefore represents a continuum of need that runs from mild to profound.

Precisely because these children are visibly vulnerable, they are highly sought after as the subject of outreach. A camp at a special school, an orphanage, or a disability institution is compassionate, photogenic, and attractive to sponsors and corporate social responsibility programmes. The optics are excellent. The difficulty is that the population is, almost by definition, one that cannot advocate for its own follow-up, and the sustained outcome, whether the child’s caries was actually treated, whether behaviour guidance was actually established, and whether recall actually happened, is rarely measured and often absent. We name this recurring pattern the *vulnerable-child optics paradox*, defined as the configuration in which the reputational and fundraising value a population confers on the provider rises as the verification of that population’s own care outcomes falls. The construct is offered as an analytic description of an incentive structure, not as an attribution of bad faith to individual programmes or volunteers, whose intentions are typically compassionate; the object of critique is the reward system, not the giver. The direct empirical warrant for the paradox is threefold: realist and empirical reviews of oral care for people with intellectual disabilities and for people with mental disorders show that sustained oral health in these groups depends on carer-mediated, relationship-based mechanisms rather than one-off contact [11,12,13]; documented camp non-attendance shows that screening contacts frequently do not convert to completed care [3]; and the Cochrane finding of very-low-certainty benefit from school screening [4] shows that visible screening activity is a poor proxy for oral health improvement. One-day charitable camps are structurally unable to supply the continuity these findings identify as necessary.

### 1.3. The Optics-to-Outcomes Inversion and the Aim of This Synthesis

Across all of these settings, children’s oral health programmes have inverted the proper relationship between visibility and impact: visibility ought to be the consequence of impact, not its substitute. The photograph should record what was achieved, yet it increasingly produces what is claimed. Two forces converge. The first is a set of sustained roadblocks, namely documented deficits in the paediatric workforce, financing, referral, surveillance, and curriculum. The second is a set of emerging distortions, namely the cultural pressures of the social media era, in which the appearance of impact has become a currency parallel to impact itself. To our knowledge, this is the first realist synthesis devoted to the optics-to-outcomes conversion problem in children’s oral health outreach; we base this on the purposive searches described in Section 2 and Supplementary Materials S1, and we make the claim of priority provisionally rather than as an exhaustive determination. It extends a small but growing realist tradition in oral health, which has so far addressed carer-led hygiene for people with intellectual disabilities [11,12], oral health for people with mental disorders [13], oral health for older people in residential aged care [14], and teledentistry implementation [15]. It organises its findings around an integrated framework, the Constellation of Care, built on three commitments: **Prevent** (acting on shared risks before disease takes hold in the child), **Promote** (closing the loop between screening and sustained care), and **Promulgate** (letting impact, evidence, and mentorship, rather than the photograph, be the visible record of the work). Throughout, general community camps and satellite outpatient services that serve all ages, including adults, are retained as the broader context within which children’s outreach sits, while the analytic emphasis remains on the child.

**Operational definitions of the three organising verbs.** Because the triad *Prevent*, *Promote*, *Promulgate* is central to the framework and one term is uncommon in health services research, each is defined operationally here. **Prevent** denotes acting on shared, upstream risks before disease is established in the child and encompasses the configurations concerned with risk-factor counselling and equity of reach. **Promote** denotes converting a detected need into completed, sustained care and encompasses the configurations concerned with navigated referral, trust brokerage, affirming clinical encounters, and interdisciplinary continuity. **Promulgate** is used in its precise sense of formally making known and putting into force, as a law or doctrine is promulgated, and is applied here to the deliberate act of making verified impact, refined programme theory, and mentored practice the authoritative, propagated record of the work. It was preferred over *implement* (which denotes carrying out a plan but not establishing it as a governing standard), over *disseminate* (which denotes spreading information and is precisely the optics behaviour the framework seeks to displace when it spreads images rather than evidence), and over *sustain* (which denotes maintaining an activity but not codifying and teaching it forward). *Promulgate* was chosen deliberately to signal that impact should be established as the enforced norm and transmitted to the next cohort through mentorship, a meaning the more conventional terms do not carry; it is retained across the manuscript with this definition.

## 2. Materials and Methods

### 2.1. Methodological Orientation

This synthesis used realist methodology as articulated by Pawson and colleagues [6,7] and was reported per the RAMESES publication standards for realist syntheses [16]. A completed RAMESES checklist is provided as part of Supplementary Materials S3. The realist orientation rests on three commitments: outcomes are produced by mechanisms triggered in contexts (the CMO configuration); the goal is to develop and refine theory rather than to estimate an average effect; and evidence is appraised on relevance and rigour rather than on methodological hierarchy alone. The RAMESES II reporting standards informed the framing of mechanisms as resource plus reasoning [17].

Throughout, mechanisms are specified as the RAMESES II couplet of a resource introduced into a context and the reasoning or response it elicits in the relevant actors. This distinction is applied consistently, including to the two antagonist configurations, so that these are read as active generative mechanisms, an introduced or absent resource that changes participant and institutional reasoning and thereby produces an outcome, rather than as passive descriptions of system gaps. Section 3.2 makes the resource and reasoning components explicit for each configuration, including CMO 7 and CMO 8.

### 2.2. Scope and Changes During Conduct

The scope was defined as community-based, time-limited oral health outreach reaching children in low- and middle-income settings, with primary emphasis on India, on school screening, and on the special care continuum, as well as with general community camps and satellite outpatient services retained as broader context. During iterative searching, the scope was widened twice to support theory development: first, to incorporate competency-based dental education, including training in paediatric and special care dentistry, as a substantive generative mechanism rather than an external factor; and second, to incorporate the institutional pressures associated with social media era reporting. Both expansions are characteristic of realist practice and are documented here per RAMESES transparency requirements.

### 2.3. Eligibility Criteria

Explicit eligibility criteria were applied and are reported in full in Supplementary Materials S2; they are summarised here. Documents were eligible when they met all of the following: (i) population predominantly children, operationally defined as aged 0–18 years, with studies of mixed child and adult populations included only where child-relevant findings or mechanisms could be separately discerned; (ii) subject matter concerning oral health outreach, defined to include school dental screening, community and anganwadi camps, mobile dental units, charitable events for children with special healthcare needs, and satellite outpatient services, together with the educational, workforce, financing, and policy conditions bearing on these; and (iii) content contributing to programme theory, meaning any inference about the contexts, mechanisms, or outcomes through which outreach did or did not convert into care. Eligible document types comprised quantitative studies, qualitative inquiries, mixed-methods studies, conceptual and methodological articles, and policy and programme reports.

Documents were excluded when they concerned adults only with no extractable child-relevant inference; when they were purely descriptive prevalence or epidemiological reports offering no contextual or mechanistic information (such reports were used, where needed, only to establish burden in the Introduction and were not treated as contributors to configuration development); when they were clinical or laboratory studies of dental materials or techniques without a public health outreach dimension; or when full text was unavailable in English. Policy documents were not held to the same appraisal template as empirical studies: they were appraised for authority, relevance, and traceability of their claims and were used to characterise the governing context rather than to evidence mechanisms. Non-peer-reviewed and grey literature (WHO and government documents and programme reports) was considered systematically where it bore on context or policy and was labelled as such, its role being to establish the policy and system environment rather than to substitute for empirical evidence of mechanisms.

### 2.4. Initial Programme Theory

An initial programme theory was developed before formal searching, drawing on the authors’ experience in dental public health and a scoping read of canonical sources (Pawson and Tilley [7]; Penchansky and Thomas [18]; Sheiham and Watt [19]; Saurman [20]; Singh and colleagues [3]; the Cochrane school-screening review [4]; and the WHO Global Oral Health Status Report [2]). Each component of the initial theory was derived from an identifiable source rather than from intuition: the access dimensions and the proposed additions of awareness and affirmation were taken from Penchansky and Thomas [18] and Saurman [20]; the common-risk-factor logic from Sheiham and Watt [19]; the screening-to-attendance gap and its drivers from Singh and colleagues [3] and the Cochrane review [4]; the burden framing from the WHO report [2]; and the CMO grammar itself from Pawson and Tilley [7]. It held that children’s camps and school screening produce sustained care when referral is specific and navigated through a caregiver or school; when all access dimensions, including affirmation, are met; when risk counselling reaches the family; when practitioners are CBE trained in paediatric and special care dentistry; when a feedback loop closes; and when institutional culture rewards verification over visibility. The theory was then exposed to the literature and iteratively refined.

**Reflexivity.** Because the initial theory drew on the team’s practitioner experience, prior assumptions could have biased interpretation toward confirming the optics-substitution thesis. This was mitigated in three ways: discordant evidence was treated as a signal of a missing contextual condition and used to revise, rather than discard, configurations; the two antagonist configurations were retained and strengthened precisely because they resist the enabling narrative; and the provenance of every configuration was recorded in the evidence-to-CMO mapping table (Supplementary Materials S3), so that readers can see which sources informed each configuration and judge the inferential distance between evidence and claim. The synthesis remains theory-generating, and the resulting configurations are presented as propositions for testing rather than as validated findings.

### 2.5. Searching Processes

Searches were purposive and iterative, in four overlapping phases. Phase 1 scoped the field. Phase 2 sought documents addressing components of the initial theory: access frameworks, the common risk factor approach, competency-based and community-based dental education, screening cascades, referral completion, and the oral health of children with special healthcare needs. Phase 3 retrieved the policy and grey literature, including WHO documents (the 2021 resolution WHA74.5, the 2022 Global Strategy WHA75.11, the 2023 Global Oral Health Action Plan WHA76.9, the 2022 Status Report, the 2024 consolidated Strategy and Action Plan, and the 2024 Bangkok Declaration), the Lancet 2019 oral health series, and Government of India sources on universal health coverage and the Viksit Bharat 2047 vision. Phase 4 tracked citations until no new configurations emerged. Databases included PubMed and MEDLINE, Embase, the Cochrane Library, Web of Science, CINAHL, and Google Scholar. No date limits were imposed; languages were restricted to English.

**Search dates, strategies, and stopping rule.** Purposive searching commenced in the scoping phase and continued through iterative theory refinement until theoretical saturation. Theoretical saturation was operationally defined as the point at which two further rounds of citation tracking and targeted searching against the candidate configurations returned no document that added a new configuration, a new contextual modifier, or a material qualification to an existing mechanism. The complete search strategies for each database, including the search strings, the dates on which each search was run, and the databases and platforms used, are provided in Supplementary Materials S1. The flow of records from identification through screening to the documents contributing to theory development, with the numbers at each stage and the reasons for exclusion, is presented as a RAMESES-consistent document-selection flow diagram in Supplementary Materials S2. Purposive sampling is retained as the appropriate realist strategy; these additions supply the methodological transparency and reproducibility the reviewers requested without converting the review into an exhaustive aggregative one.

### 2.6. Selection, Appraisal, Extraction, and Analysis

Documents were appraised on relevance (contribution to theory) and rigour (credibility of inference) rather than on a fixed hierarchy [16]. Quantitative studies, qualitative inquiries, policy documents, programme reports, and conceptual articles were all admitted. From each retained document, bibliographic details, study type, setting, population, outcomes, and, critically, any inference about how, why, or under what conditions the intervention worked or failed were extracted and tagged provisionally to candidate configurations. Analysis clustered the extracted material into candidate CMOs, refined them through juxtaposition, reconciliation, adjudication, and consolidation across documents [7], treated discordance as a clue to a missing contextual condition rather than a reason to abandon a configuration, and reintegrated the consolidated CMOs into a refined programme theory. The five cross-cutting modifiers emerged through this process. The mapping of which documents informed each configuration is reported in the evidence-to-CMO table (Supplementary Materials S3).

## 3. Results

### 3.1. Document Flow and Cross-Cutting Modifiers

The iterative search identified a large background corpus, of which thirty-six documents were retained as the full set of principal contributors to programme theory (the same 36 documents that constitute the reference list of this article); these are the documents that materially shaped one or more configurations or modifiers, as distinct from the wider background corpus consulted during scoping but not cited. They span realist methodology, access theory, the common risk factor approach, empirical studies of screening and referral, the paediatric caries burden, the oral health of children with special healthcare needs, competency-based and community-based dental education, workforce and financing analyses, and WHO and Government of India policy. Five macro-level modifiers, invoked repeatedly across the corpus, calibrate the activation of every mechanism: the paediatric skill mix of the workforce; the financing model; school and caregiver mediation; the reporting and visibility culture; and the receiving paediatric care system. The workforce modifier is sharp: country income status and urbanisation, more than absolute numbers, predict where dentists actually deliver care, with maldistribution identified as the central challenge for lower-income countries [21], and qualitative inquiry with Indian public health dentists frames this as a distribution rather than a shortage problem, locating the discontinuity between detection and definitive care as structural [22].

### 3.2. The Eight CMO Configurations

**Figure 1:**
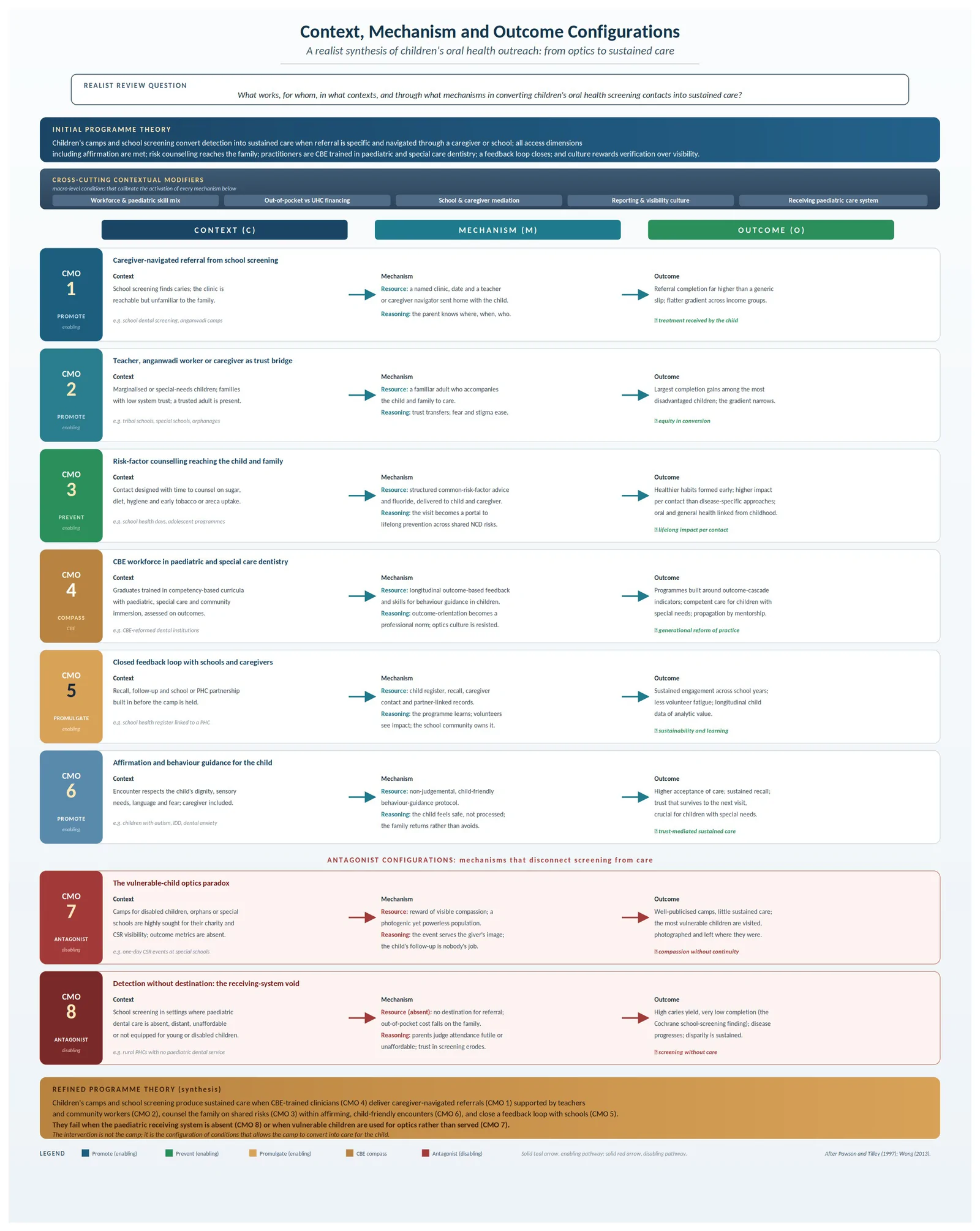
Context, Mechanism, and Outcome configuration framework for children’s oral health outreach. The Initial Programme Theory and cross-cutting modifiers frame eight configurations, six enabling (CMO 1 to 6) and two antagonistic (CMO 7 and 8). The Refined Programme Theory integrates them: the intervention is not the camp; it is the configuration of conditions that allows the camp to convert into care for the child (Pawson and Tilley (1997) [7], Wong (2013) [16]).

The synthesis yielded eight configurations, six enabling and two antagonistic, summarised in Figure 1 and narrated below. For each, the mechanism is stated as an introduced or absent resource and the reasoning it triggers, per RAMESES II [17].

#### 3.2.1. CMO 1: Caregiver-Navigated Referral from School Screening (Promote)

Where school screening detects caries and the clinic is reachable but unfamiliar to the family, providing a named clinic, a date, and a teacher or caregiver who carries the referral home (resource) activates reduced cognitive load and increased caregiver self-efficacy (reasoning): the referral acquires a face and a date. The outcome is referral completion far higher than a generic slip achieves and a flatter gradient across income groups, consistent with Singh and colleagues’ finding that non-attendance was driven by operational uncertainty rather than refusal [3].

#### 3.2.2. CMO 2: Teacher, Anganwadi Worker, or Caregiver as Trust Bridge (Promote)

For children who are marginalised or with special needs whose families hold low trust in the health system, a familiar adult who shares language, culture, and social position and who accompanies the child and family to care (resource) activates the transfer of trust from a known figure to an unfamiliar facility, easing fear and stigma (reasoning). The outcome is the largest completion gains among the most disadvantaged children, so that the access gradient narrows. This is supported by qualitative inquiry into the Indian landscape [22] and by the social accountability tradition in dental education [23,24].

#### 3.2.3. CMO 3: Risk-Factor Counselling Reaching the Child and Family (Prevent)

Where the contact is designed with time to counsel the child and caregiver on sugar, diet, hygiene, fluoride, and the early uptake of tobacco or areca (resource), dental trust generalises to general health advice, and the visit becomes a portal to lifelong prevention across shared noncommunicable disease risks (reasoning). The outcome is healthier habits formed early and a higher impact per contact than disease-specific approaches achieve, drawing on the common risk factor approach [19,25] and its later social-determinants articulation [26] and aligning with the WHO strategy’s primary-care integration objective [27].

#### 3.2.4. CMO 4: CBE Workforce in Paediatric and Special Care Dentistry (Compass)

Where graduates have trained under competency-based curricula with paediatric, special care, and community immersion, assessed on outcomes (resource), longitudinal exposure to outcome-based feedback internalises outcome orientation as a professional norm and builds the behaviour-guidance skills that young children and those with disabilities require (reasoning). The outcome is the design of programmes around outcome-cascade indicators, competent care for children with special needs, and propagation of the model to the next cohort through mentorship. Community-based dental education is associated with gains in student confidence, cultural competence, professional identity, and readiness for population-oriented practice [23,24,28]. CBE is positioned here not as one component among others but as the generative mechanism by which the other enabling configurations come reliably to be activated.

#### 3.2.5. CMO 5: Closed Feedback Loop with Schools and Caregivers (Promulgate)

Where recall, follow-up, and school or primary care partnership are designed in before the camp is held (resource), the programme becomes a learning system (reasoning): the team perceives evidence of its own impact, which sustains motivation against volunteer fatigue; the school community perceives continuity rather than visitation, which sustains trust; and the institution acquires longitudinal child data of analytic value. The outcome is sustained engagement across school years, reduced volunteer fatigue, and community ownership, the elements that constitute genuine sustainability rather than mere repetition.

#### 3.2.6. CMO 6: Affirmation and Behaviour Guidance for the Child (Promote)

Where the encounter respects the child’s dignity, sensory needs, language, and fear and includes the caregiver (resource), the child feels safe rather than processed, and the family returns rather than avoids (reasoning). The outcome is higher acceptance of care, sustained recall, and trust that survives to the next visit, an outcome that is decisive for children with autism, intellectual and developmental disability, or dental anxiety. Affirmation is grounded in the Penchansky and Thomas access framework [18] as extended by Saurman’s addition of awareness as a sixth dimension [20], with affirmation proposed here as a seventh, and it is the everyday clinical expression of the carer-mediated mechanisms that realist reviews identify as central to special care [11,12].

#### 3.2.7. CMO 7: The Vulnerable-Child Optics Paradox (Antagonist)

Where feedback arrives principally through visibility channels and where the population is photogenic yet unable to advocate for its own follow-up, as with camps at special schools, orphanages, and disability institutions, the operative resource is the reputational and fundraising reward supplied to the provider by visible compassion; the reasoning it triggers is that this reward is readily accepted as a proxy for impact, which lowers the perceived need to verify follow-up. The mechanism is structural rather than motivational: it describes how an incentive that rewards visibility, in the absence of any requirement to report outcomes, shifts attention away from follow-up, and it does not depend on any assumption about the intentions of the individuals involved. This is an active generative mechanism, not merely a gap: the introduced resource changes what participants and institutions attend to and thereby produces the outcome. The child’s follow-up becomes nobody’s responsibility. Expressed in structural terms, no actor in the configuration holds a defined responsibility for verifying the child’s follow-up, and no mechanism records whether it occurred. The outcome is well-publicised camps that deliver little sustained care, in which the most vulnerable children are screened without a documented pathway to completed treatment or recall. This is the antagonist that the special care continuum makes most visible, and it is why one-off charitable events, whatever their intent, cannot substitute for a relationship [11,13].

#### 3.2.8. CMO 8: Detection Without Destination, the Receiving-System Void (Antagonist)

Where school screening occurs in settings in which paediatric dental care is absent, distant, unaffordable, or not equipped for young children or those with disabilities, the operative mechanism is the absence of a resource, a reachable and appropriate destination for referral, which triggers a specific caregiver reasoning: a local cost–benefit calculation that attendance is futile or unaffordable, so that effort is withheld and trust in screening erodes. Framed this way, CMO 8 is not a passive systemic deficiency but a mechanism because the missing resource actively reshapes caregiver reasoning and produces the outcome. The structural condition is common across rural India and under out-of-pocket-dominant financing [2]. The outcome is high caries yield with very low completion, the classic school-screening finding [4], alongside iatrogenic mistrust that may degrade the next encounter and a disparity that is thereby sustained [3], a burden that falls hardest on children with special healthcare needs, of whom a large share need dental services but cannot obtain them [10].

### 3.3. The Refined Programme Theory

Children’s camps and school screening produce sustained care when CBE-trained clinicians (CMO 4) deliver caregiver-navigated referrals (CMO 1) supported by teachers and community workers (CMO 2), counsel the family on shared risks (CMO 3) within affirming, child-friendly encounters (CMO 6), and close a feedback loop with schools (CMO 5). They fail when the paediatric receiving system is absent (CMO 8) or when outreach to vulnerable children generates visibility without a structured pathway to verified follow-up (CMO 7).

The intervention is not the camp; it is the configuration of conditions that allows the camp to convert into care for the child.

Three corollaries follow. First, no single configuration is sufficient: their effects are configurational, and the absence of CMO 4 reliably attenuates the activation of CMOs 1 to 3, 5, and 6. We note that “reliably attenuates” is a theoretical proposition generated by this synthesis, consistent with the assembled evidence but not yet quantified; it is advanced as a hypothesis for prospective realist evaluation rather than as an established effect. Second, the antagonist configurations operate regardless of whether enabling configurations are present because they sit closer to institutional and structural roots. Third, the cross-cutting modifiers calibrate every configuration, so that a pattern performing well under one financing model or curricular paradigm may not transfer to another.

### 3.4. The Constellation of Care: Organising the Synthesis

**Figure 2:**
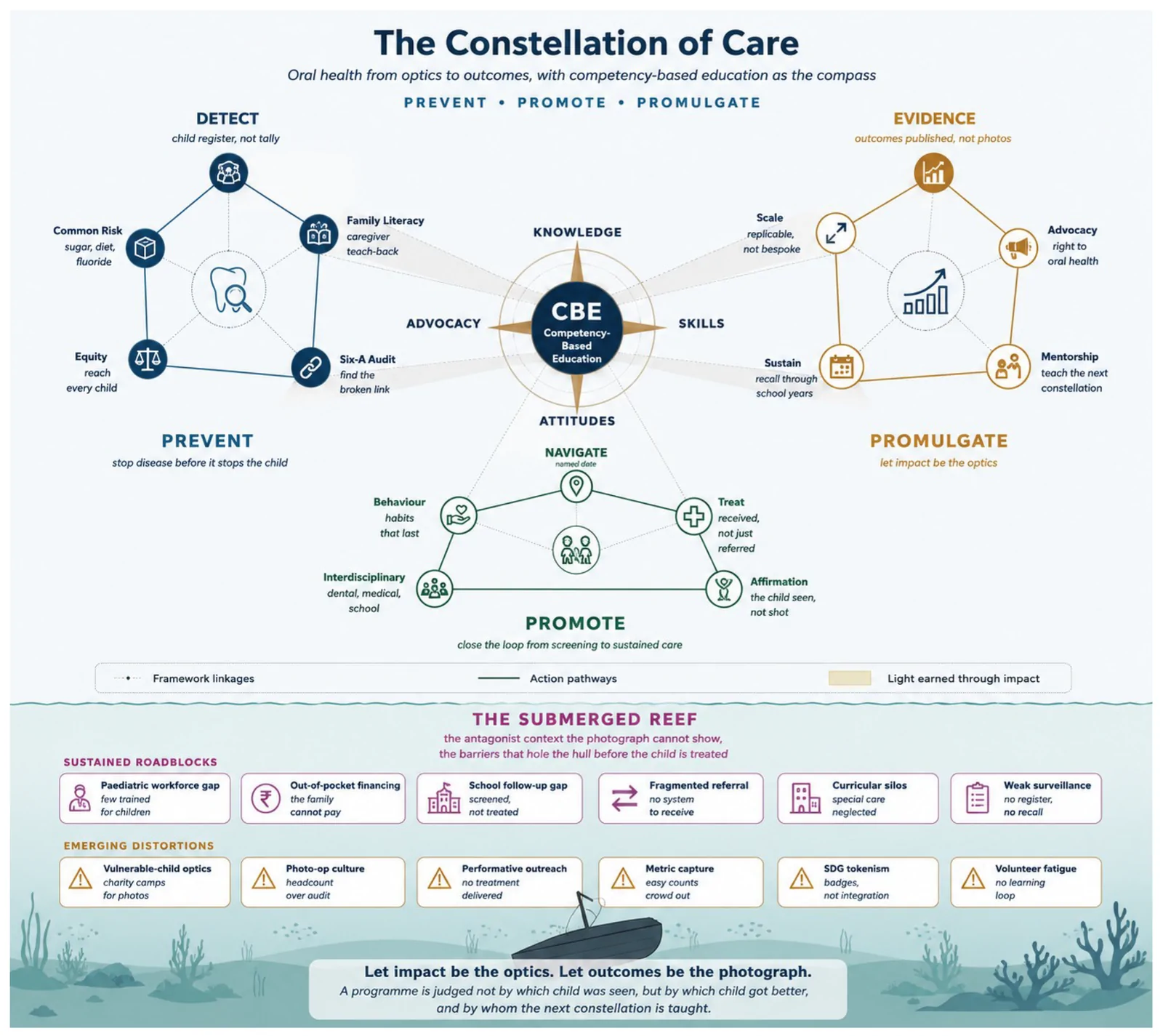
The Constellation of Care. Three thematic constellations—Prevent (**left**), Promulgate (**right**), and Promote (**bottom**)—are navigated by a central compass of competency-based education whose cardinal axes are the four CBE domains. Dotted lines are framework linkages, and solid lines are action pathways; the shaded band marks light earned through impact. The optics threshold separates the visible surface of impact from the submerged reef of antagonist context, where the wreck of the one-day charity camp lies. Expanded legend for interpretation: each labelled “star” is a theoretical construct proposed by this synthesis, not a discrete empirical finding; solid pathways denote proposed causal action routes derived from the CMO configurations, whereas dotted links denote conceptual relationships within the framework. Constructs above the optics threshold represent verifiable impact, and elements below it represent the antagonist contexts (CMO 7 and CMO 8). Readers seeking the empirical basis for any construct are referred to the evidence-to-CMO mapping in Supplementary Materials S3, which distinguishes empirically grounded elements from theoretical connective tissue.

The eight configurations are organised conceptually into three thematic constellations navigated by a central compass of competency-based education (Figure 2). **Prevent** gathers the upstream stars (Detect, Common Risk, Family Literacy, Equity, and Six-A Audit); **Promote** gathers the conversion stars that close the screening-to-care loop (Navigate, Treat, Behaviour, Interdisciplinary, and Affirmation); and **Promulgate** gathers the downstream stars that make impact the visible record (Evidence, Advocacy, Scale, Mentorship, and Sustain). The compass’s four cardinal points—Knowledge, Skills, Attitudes, and Advocacy—are the CBE domains that orient movement through every star. An explicit optics threshold separates the visible sky of impact from the submerged reef of sustained roadblocks and emerging distortions, including the paediatric workforce gap, the school follow-up gap, neglected special care, and the vulnerable-child optics that this synthesis foregrounds. The SDGs form an interconnected web threaded across all three constellations.

## 4. Discussion

### 4.1. What Works, for Which Children, Why, and in What Conditions

Children’s camps and school screening work, in the meaningful sense of producing sustained care, not because they are camps but because in particular configurations they activate the causal architecture by which detection converts into care. They work most decisively for socially marginalised children, as well as for children with special healthcare needs, when CMO 2 (trusted navigator) and CMO 6 (affirmation and behaviour guidance) operate; they work because trust earned in the encounter generalises to the navigation of unfamiliar systems and to the acceptance of broader health advice by the family; and they work under conditions that include a paediatric receiving system to which referral can terminate, a workforce educated to value outcome over activity, and an institutional culture that does not reward the photograph over the child.

**Direct evidence versus interpretation.** It is important to separate what the evidence shows from what this synthesis infers. Directly evidenced are the following: camp-based screening is frequently followed by low referral completion [3]; school screening has very-low-certainty benefit for attendance and unproven benefit for oral health [4]; sustained oral health in populations with intellectual disabilities and mental unwellness depends on continuous, carer-mediated care rather than episodic contact [11,12,13,14]; community-based dental education is associated with measurable gains in graduate disposition [23,24,28]; and workforce maldistribution, not absolute scarcity, is the central lower-income-country constraint [21]. The claims that these findings are best explained by the eight configurations, that CBE is the generative lever, and that affirmation constitutes a distinct access dimension are the authors’ realist interpretation, offered as theory to be tested. Throughout the Discussion, we have tried to keep this boundary visible.

A related distinction concerns the provenance of the supporting evidence. Several sources that inform the configurations are not drawn directly from paediatric oral health outreach: the realist and empirical work on carer-mediated continuity concerns adults with intellectual disability, people with mental disorders, and older people in residential aged care [11,12,13,14], and the education evidence concerns dental students and graduates rather than child outcomes [23,24,28]. In realist synthesis, such transfer is legitimate because the object of interest is the mechanism (here, that continuity of a trusted, carer-mediated relationship sustains oral health) rather than the specific population in which it was first observed. The transfer is nonetheless an inferential step, and it is treated as such here: where a configuration rests on evidence transferred from an adjacent population, the supporting mechanism is advanced as a working proposition whose transportability to children’s outreach requires confirmation, rather than as a directly demonstrated finding. The evidence-to-CMO mapping in Supplementary Materials S3 records, for each configuration, whether its supporting sources are drawn from children’s outreach directly or transferred from an adjacent field, so that the strength and directness of the underlying evidence can be judged configuration by configuration.

### 4.2. The Special Care Continuum, from Charity to Continuity

The special care continuum sharpens every argument in this synthesis. Children with special healthcare needs carry a heavier oral disease burden, face steeper access barriers, and report dental care as their most prevalent unmet health need, with unmet need rising as the severity of the condition rises [5,8,9,10]. They are also, for the same reasons that make them vulnerable, a population for which outreach carries high visibility. The vulnerablechild optics paradox (CMO 7) is therefore not a marginal case but a lens that reveals the general failure with unusual clarity: a configuration in which a highly visible event is not structurally coupled to any process that verifies the child’s subsequent care. The corrective is not to stop serving these children but to change what counts as service. Realist and empirical work in intellectual disability, mental disorder, and residential care converges on a single message: oral health in these groups is produced by continuous, carer-mediated, relationship-based care, not by episodic contact [11,12,13,14], which is exactly the continuity that CMO 5 (closed feedback loop) and CMO 6 (affirmation) are designed to supply.

### 4.3. The Competency-Based Education Imperative and What Is Achievable Sooner

Among the eight configurations, CMO 4 is the only one whose activation generationally reorients the field. Curricular reform is the slowest and most determinative lever in any health system: slow because it acts on cohorts and determinative because it shapes the assumptions a generation carries into practice. The community-based dental education literature documents that explicit, outcome-based, community-immersed education produces graduates measurably different in professional disposition from those trained under legacy curricula [23,24,28]. Three integration points are tractable: redesigning community and school postings so that students are assessed on referral completion rather than camps conducted; embedding paediatric behaviour guidance and access-dimension auditing as assessed clinical competencies, with explicit training in special care dentistry; and requiring postgraduate research to report the referral completion rate of any programme studied. This is the mechanism by which *Promulgate* ceases to be a slogan and becomes a curricular outcome.

Because full curricular reform is slow, the framework does not depend on it in the short term. Several measures are achievable within existing structures and timeframes. In the short term (institution-led, within one to two academic cycles), these are as follows: adopt a named-child register and an outcome-cascade reporting template for every outreach event; add referral completion follow-up as a mandatory element of existing community postings without changing the formal curriculum; and require postgraduate dissertations to report referral completion as an outcome. In the medium term (department- and regulator-facing), these are as follows: incorporate outcome-cascade reporting into ethics committee and sponsor templates; add short continuing-education modules on paediatric behaviour guidance and special care for existing practitioners, which reach the current workforce without waiting for a new graduate cohort; and pilot the framework in a small number of partner schools. Formal competency-based curricular change is then the long-term consolidation of gains already demonstrable through these earlier steps.

### 4.4. The SDG Web: Never a Badge, Always a Weave

**Figure 3:**
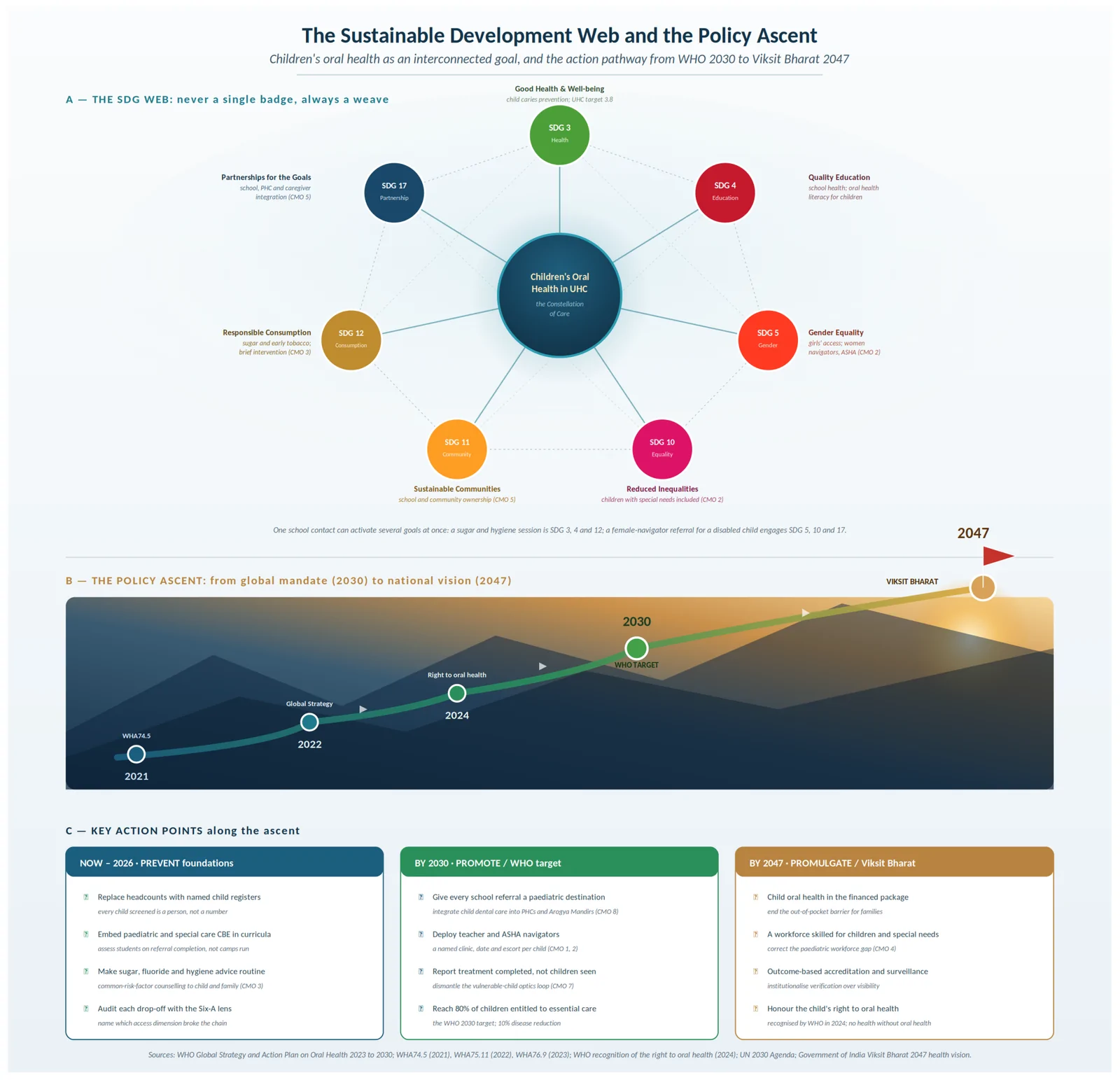
The Sustainable Development web and the policy ascent for children’s oral health. Panel (**A**): children’s oral health within universal health coverage as an interconnected SDG node. Panel (**B**): the ascent from the 2021 World Health Assembly resolution through the 2022 Global Strategy and the 2024 recognition of the right to oral health to the WHO 2030 target and India’s Viksit Bharat 2047 vision. Panel (**C**): key action points arranged by horizon and by the Prevent, Promote, and Promulgate sequence.

The goals of the 2030 Agenda are explicitly integrated and indivisible [29]. Children’s oral health touches at least eight of them directly, and programmes structured by the configurations identified here engage them as a web rather than a list (Figure 3, Panel A). A single school session that activates CMO 3 on sugar and hygiene contributes simultaneously to SDG 3 (health), SDG 4 (education through literacy), and SDG 12 (responsible consumption); a female-navigator referral for a child with disabilities under CMO 2 engages SDG 5 (gender), SDG 10 (reduced inequalities), and SDG 17 (partnerships); and a closed feedback loop with a school under CMO 5 engages SDG 11 (sustainable communities). Reporting that articulates the weave, rather than badging the single goal claimed, disciplines design and resists the SDG tokenism documented as an emerging distortion [30].

### 4.5. The Policy Ascent: WHO 2030, the Right to Oral Health, and Viksit Bharat 2047

The framework is positioned within the prevailing policy architecture (Figure 3, Panel B). At the global level, the 2021 resolution (WHA74.5) recognised oral health as a priority within the noncommunicable disease agenda; the 2022 Global Strategy (WHA75.11) set the vision of universal health coverage for oral health by 2030; the 2023 Action Plan (WHA76.9), consolidated in 2024, translated this into six objectives, eleven targets, and one hundred actions; and in 2024, the WHO recognised the right to oral health and Member States adopted the Bangkok Declaration, No Health Without Oral Health [27,31,32,33]. The overarching 2030 target is that 80% of the global population be entitled to essential oral health services, with a 10% relative reduction in the main oral diseases, aligned with SDG target 3.8. For children, this recognition of a right to oral health is more than rhetorical: it reframes the untreated caries of half a billion children as a matter of entitlement rather than charity, and it places the burden of proof on programmes to show that a child was served, not merely seen.

At the national level, India’s Viksit Bharat 2047 vision, the aspiration to become a developed nation by the centenary of independence, positions universal health coverage as a foundational pillar, advanced through the Ayushman Bharat Pradhan Mantri Jan Arogya Yojana and the network of Ayushman Arogya Mandirs that deliver primary care at the grassroots [34,35]. Yet two features of this landscape map onto the antagonist configurations of this synthesis. First, oral health, and children’s oral health in particular, remains largely outside the publicly financed benefit package, and out-of-pocket payment continues to push tens of millions into poverty each year [35], which is the financing face of CMO 8. Second, the receiving-system void that disables outreach is precisely what primary care integration through Arogya Mandirs is intended to fill; where that integration omits paediatric dental care, referral continues to terminate in absence. The Constellation of Care engages this scheme directly: CMO 8 names the gap that integration must close, CMO 7 names the reporting culture that national programmes must resist, and CMO 4 is the human resource engine common to the WHO 2030 target and the Viksit Bharat 2047 vision alike. As the Bangkok Declaration holds, there is no health without oral health [33], and, as the national vision holds, only a healthy nation can be a developed nation.

### 4.6. Beyond National Income: Health System Capacity, Utilisation, and the Outreach-to-Care Continuum

A further insight emerging from this synthesis is that the success or failure of children’s oral health outreach cannot be adequately explained by a country’s economic status alone. Conventional narratives frequently position low- and middle-income countries (LMICs) as resource-constrained systems and high-income countries (HICs) as models of comprehensive healthcare delivery. The evidence synthesised here suggests a more nuanced reality. The decisive determinant is not national wealth per se but the ability of a health system to activate and sustain the mechanisms that convert screening into definitive care, prevention into behavioural change, and outreach into measurable health outcomes.

Many LMICs, including India and several Southeast Asian nations, continue to face a disproportionately high burden of childhood oral disease, driven by demographic pressures, socioeconomic inequalities, workforce maldistribution, and substantial unmet treatment needs [1,21]. Yet these same settings have progressively invested in expansive community-based public health infrastructure through school health programmes, anganwadi services, frontline health workers, mobile dental units, primary healthcare reforms, and large-scale governmental outreach initiatives. Such platforms create repeated opportunities to engage children and families who might otherwise remain outside formal healthcare systems. The critical challenge, therefore, is no longer the absence of outreach but ensuring that these encounters culminate in completed referrals, timely treatment, sustained preventive behaviours, and longitudinal follow-up. Within the present synthesis, this distinction separates programmes that merely demonstrate activity from those that genuinely demonstrate impact.

Conversely, high-income countries illustrate that financial prosperity and advanced healthcare infrastructure do not automatically guarantee equitable oral healthcare access or successful care pathways. Despite comparatively greater workforce density, specialist availability, and insurance coverage, dental care remains among the most frequently reported unmet healthcare needs for children, particularly those with special healthcare needs and socially disadvantaged populations [5,9,10]. In these settings, discontinuities often arise from fragmented service delivery, inadequate integration between screening and treatment services, workforce maldistribution, prolonged waiting times, insurance limitations, and failures of care coordination. Consequently, the antagonist configuration of Detection without Destination (CMO 8) transcends economic classification; it represents a systems-level failure that emerges whenever outreach identifies disease without ensuring an accessible, acceptable, affordable, and appropriate pathway to definitive care.

Viewed through a realist lens, these observations reaffirm that health system performance is fundamentally configurational rather than economic. Resource availability undoubtedly shapes context, but resources alone do not trigger successful outcomes. Instead, outcomes emerge when contextual conditions activate mechanisms such as trusted community navigation, caregiver engagement, competency-based professional education, child-centred communication, and closed feedback loops that collectively sustain the continuum from detection to treatment. Wealth may increase the availability of services, but only effective integration determines whether those services translate into improved health. Where governments have invested substantially in outreach, the emphasis should shift from measuring programme reach to measuring utilisation and outcome verification, since camps organised, children screened, or awareness sessions conducted represent only the beginning of the care pathway, whereas referral completion, treatment initiation and completion, behavioural maintenance, recall attendance, and sustained oral health improvement are the meaningful indicators.

### 4.7. Stakeholder Perspectives and Testability in Real-World Settings

This synthesis was conducted without formal stakeholder consultation, which is a limitation (Section 4.9). Anticipating how key actors would view the model is nonetheless useful for its refinement. Caregivers and teachers, on whom CMO 1, CMO 2, and CMO 6 depend, are likely to value the shift from being counted to being supported but may reasonably ask who bears the added navigation workload. Frontline dentists and volunteers may welcome outcome-cascade reporting as recognition of real work yet may resist it where it adds documentation without added resources. Policymakers and sponsors, addressed by CMO 5, CMO 7, and CMO 8, control the reporting templates and financing that the framework asks to change, and their incentives are the ones the antagonist configurations describe. A prospective realist evaluation is the natural test: a small number of outreach programmes could be configured to activate the enabling CMOs (named-child registers, navigator support, and designed-in recall) and compared, on referral completion and treatment completion, with conventionally reported programmes, with qualitative interviews used to confirm or revise the proposed mechanisms and to surface stakeholder-identified configurations the present theory may have missed. Such an evaluation would move the eight configurations from proposition toward testing.

### 4.8. Comparison with Previous Literature

This synthesis locates the Singh and colleagues finding of camp non-compliance [3] as the canonical demonstration of CMO 8; situates the Cochrane review’s very-low-certainty finding [4] within a causal architecture in which screening is not the unit of causation, the configuration is; and extends the Penchansky and Thomas and Saurman access tradition [18,20] by proposing affirmation as a distinct access dimension for children. It is consonant with the Lancet 2019 series’ diagnosis of the global neglect of oral health [36], with the workforce analysis identifying maldistribution as the central challenge for lower-income countries [21], and with the paediatric and special care literature on the unmet needs of children generally and children with special healthcare needs in particular [1,8,9,10].

### 4.9. Strengths and Limitations

**Strengths.** The synthesis follows the RAMESES standards [16], is, to our knowledge, the first realist synthesis of the optics-to-outcomes problem in children’s oral health, draws on the empirical, qualitative, conceptual, and policy literature, articulates a causal account testable by prospective realist evaluation, and integrates antagonist mechanisms that conventional reviews leave as contextual nuisance.

**Limitations.** The synthesis was conducted by a single research team without formal stakeholder consultation. Its geographic emphasis is Indian and South Asian, and the configurations should be validated against other settings before generalisation. Realist synthesis produces theory rather than effect estimates, so the configurations are to be tested by prospective realist evaluation [17] rather than treated as confirmed causal claims. The literature on the antagonist configurations is thinner than that on the enabling ones, which is itself an artefact of the optics culture under critique. The initial programme theory was informed by the team’s practitioner experience; the reflexive safeguards in Section 2.4 mitigate but do not eliminate the associated risk of confirmatory interpretation. A further limitation is that part of the evidence base is indirect: several configurations rest on mechanisms transferred from adjacent populations and from the education literature rather than from paediatric oral health outreach itself (Section 4.1). This reflects the current scarcity of outcome-level evidence in children’s outreach, and it reinforces that the configurations are hypotheses for direct empirical testing rather than established findings.

### 4.10. Implications Across Levels

- **Practice (the contact).** Operationalise CMOs 1 to 3 and 6: name the clinic and date; send a teacher or caregiver navigator with the child; deliver risk-factor counselling to child and family as default; conduct every encounter with attention to the child’s dignity, sensory needs, and fear; and replace the tally with a named-child register.
- **Programme.** Operationalise CMO 5: design recall and follow-up before the camp is held; partner with schools, anganwadis, and primary care; and report the outcome cascade (screened, referred, treated, and sustained), not the headcount.
- **Education.** Operationalise CMO 4: reorient community and school postings, paediatric and special care teaching, and postgraduate research around outcome verification; make behaviour guidance and access-dimension auditing assessed competencies.
- **Policy.** Neutralise CMOs 7 and 8: require outcome cascades in accreditation and sponsor templates; integrate paediatric dental care into primary care and Arogya Mandirs so that referral has a destination; and align programme evidence with the WHO Global Strategy [27,31], the 2024 recognition of the right to oral health [33], and the Viksit Bharat 2047 agenda [34].

### 4.11. An Outcome-Cascade Indicator Framework

Table 1 operationalises this shift into an outcome-cascade indicator framework, contrasting, at each stage of the care pathway, the optics indicator that is typically counted with the verifiable outcome indicator that should replace it, together with an illustrative verification method and proposed target. Basis and status of the proposed targets. The numerical values in the target column are expert-derived, proposed programme-design aspirations, not evidence-based standards or validated thresholds. They were set by author consensus to represent a meaningful advance over the low completion rates documented for camp-based screening [3,4] and are offered so that programmes have a concrete starting point to adapt to the local baseline, capacity, and context. They should be calibrated against local data and are expected to differ between settings; they must not be cited as established benchmarks. Programmes that prefer to avoid numerical anchors may use the outcome domains alone and set their own locally justified targets.

## 5. Conclusions

Children’s oral health programmes have for too long optimised for what the camera can see. This realist synthesis argues that they should optimise instead for what the child can feel, namely a completed treatment, a reduced risk, and a sustained change, and it offers a causal account, expressed as eight configurations and organised as the Constellation of Care, of when and why this conversion succeeds or fails. It relocates the intervention from the camp to the configuration of conditions that allows the camp to convert into care for the child, and it identifies competency-based education, in paediatric and special care dentistry, as the generative mechanism by which this configuration becomes the default rather than the exception. The special care continuum shows the stakes most plainly: the children most useful for a photograph are the children most in need of a relationship. These configurations are theoretical propositions generated by synthesis, not confirmed causal claims; they now require prospective empirical testing through realist evaluation, and ideally with stakeholder involvement, before they are adopted as established. The proposition is therefore simple and demanding in equal measure that a programme should be judged not by which child was seen but by which child got better and by whom the next constellation is taught.

## Figures and Tables

**Table 1:** From optics to outcomes: an outcome-cascade indicator framework for children’s oral health outreach, with illustrative verification methods and expert-derived, proposed (non-standard) targets.

Care Pathway Stage	Optics Indicator (Typically Counted)	Outcome Indicator (To Be Verified)	Verification Method or Data Source	Proposed Target (Expert-Derived; Adapt Locally)	Config. and Domain
Reach and mobilisa-tion	Camps held; children screened; awareness sessions delivered	Proportion of eligible children reached; reach among the poorest children and children with special-needs	School and anganwadi rolls as denominator; disaggregated attendance register	≥80% of enrolled children reached; no subgroup below 70%	CMO 2; Prevent, Promote
Detection and risk assessment	Children examined; aggregate caries prevalence reported	Named-child register with individual caries status and caries-risk category	WHO oral health assessment form completed per child; risk categorised	100% of examined children entered in a recallable register	CMO 1; Prevent
Caregiver-navigated referral	Referral slips issued	Referral completion rate; the child reached a named facility	Two-way referral card matched to facility attendance record	≥60% referral completion (against the low rates reported for camps)	CMOs 1, 2, 8; Promote
Definitive treatment	Treatment reported as provided at the camp	Treatment initiation and completion; time to definitive care	Treatment log linked to the child register; date of completion recorded	≥80% of detected treatment needs completed within three months	CMOs 4, 8; Promote
Prevention and behaviour change	Oral-hygiene talks delivered; leaflets distributed	Caregiver teach-back achieved; behaviour maintained at follow-up	Teach-back checklist; plaque and diet indicators recorded at recall	≥70% of caregivers demonstrate correct technique at follow-up	CMOs 3, 6; Prevent, Promote
Continuity, recall, and follow-up	Repeat camp conducted	Recall attendance; longitudinal change in oral health status	Scheduled recall through school or PHC; repeat examination at intervals	≥75% recall attendance sustained across a school year	CMO 5; Promulgate
Equity and special care	Charity camp at a special school photographed	Individual sustained-care plan per child with special healthcare needs; unmet-need gap closed	Individual care plan; unmet-need audit disaggregated by disability and disadvantage	Unmet-need gap for CSHCN no wider than for peers	CMOs 2, 6, 7; Promote
Dissemination and learning	Photographs, press coverage and social media reach	Outcome cascade published; programme theory refined and taught to the next cohort	Public outcome report; CMO-based evaluation; documented mentee cohort	Full cascade, from screened to sustained, reported each programme cycle	CMO 5; Promulgate

CMO, Context–Mechanism–Outcome configuration (Figure 1); CSHCN, children with special healthcare needs; PHC, primary health centre. Optics indicators record activity and visibility, whereas outcome indicators verify that a child received and sustained care. Targets are expert-derived, proposed programme-design aspirations, not established standards, and must be adapted to local baseline and context.

## Data Availability

No new data were created or analysed in this study. Data sharing is not applicable to this article.

## Supplementary Materials

The following supporting information can be downloaded at https://www.mdpi.com/article/10.3390/children13091212/s1, Supplementary Materials S1. Database Search Strategies, Strings and Dates; Supplementary Materials S2. Inclusion/Exclusion Criteria and Document-Selection Flow; Supplementary Materials S3. Evidence-to-CMO Mapping and RAMESES Checklist.

## Abbreviations

The following abbreviations are used in this manuscript:

CBE CMO	Competency-Based Education Context, Mechanism, and Outcome
CSHCN	Children with Special Healthcare Needs
HIC	High-Income Country
LMIC	Low- and Middle-Income Country
PHC	Primary Health Centre
RAMESES	Realist And Meta-narrative Evidence Syntheses: Evolving Standards
SDG	Sustainable Development Goal
WHO	World Health Organization
