# Supplementary material for: Prevent, Promote, Promulgate®: A Realist Synthesis of Oral Health Outreach from Optics to Outcomes, Across Children, Communities, and the Special Care Continuum
Source: Children (Basel). 2026 Sep 8;13(9):1212. doi: 10.3390/children13091212 (PMC13604955; doi:10.3390/children13091212)
Supplement: Supplementary file 1 [file children-13-01212-s001.zip › children-4496292-supplementary.pdf]

Supplementary Materials

Prevent, Promote, Promulgate: A Realist Synthesis of Oral Health Outreach from Optics to Outcomes, Across Children, Communities and the Special Care Continuum

Vaibhav Kumar, Praveen Jodalli, Ziad D. Baghdadi, Ramya Shenoy, Ishan Maliye and Ridhima Gaunkar

Submitted to Children (MDPI). This file contains Supplementary Materials S1–S3 referenced in the main text.

This document provides the supporting methodological detail requested by the reviewers. It comprises three parts: **S1** the complete database search strategies, strings and dates; **S2** the inclusion/exclusion criteria and the RAMESES-consistent document-selection flow diagram; and **S3** the evidence-to-CMO mapping table and the completed RAMESES publication-standards checklist. Consistent with realist methodology, searching was purposive and iterative; the numeric counts reported here document that process transparently and are not intended to imply an exhaustive aggregative search.

Supplementary Materials S1. Database Search Strategies, Strings and Dates

Searches were conducted between **15 January 2025 and 10 August 2025**, with a final citation-tracking and verification pass completed on **22 August 2025**. Six bibliographic databases and one general scholarly index were searched. No date limits were applied; results were restricted to English-language full texts. Search concepts were combined across four blocks: (A) children/adolescents; (B) oral/dental health; (C) outreach, screening, camps and referral; and (D) the theory-relevant blocks added during iterative refinement (access, common risk factor, competency-/community-based education, special care, and social-media/reporting culture). Block D terms were introduced progressively as programme theory developed, in line with realist practice.

S1.1. Concept blocks (controlled vocabulary and free text)

Concept block	Search terms (representative; adapted to each database syntax)
A. Children / adolescents	child*, children, paediatric, pediatric, adolescen*, school-age*, “school child*”, anganwadi; MeSH: Child, Adolescent, Child, Preschool, Schools
B. Oral / dental health	“oral health”, dental, caries, “dental caries”, “oral disease”; MeSH: Oral Health, Dental Caries, Dental Health Services
C. Outreach / screening / referral	outreach, “screening programme”, “school dental screening”, camp*, “mobile dental”, referral, “referral completion”, “non-attendance”, “community dental”; MeSH: Mass Screening, Community Dentistry, Referral and Consultation
D. Theory blocks (iterative)	“access to care”, “common risk factor”, “competency-based education”, “community-based education”, “special care dentistry”, “special health care needs”, “intellectual disability”, realist, RAMESES, “social media”, “reporting culture”; MeSH: Health Services Accessibility, Education, Dental, Disabled Children

S1.2. Database-specific strategies

PubMed / MEDLINE (searched 15–18 Jan 2025; updated 8 Aug 2025)

((child*[tiab] OR children[tiab] OR paediatric[tiab] OR pediatric[tiab] OR adolescen*[tiab] OR “Child”[Mesh] OR “Adolescent”[Mesh]) AND (“oral health”[tiab] OR dental[tiab] OR caries[tiab] OR “Oral Health”[Mesh] OR “Dental Caries”[Mesh]) AND (outreach[tiab] OR screening[tiab] OR camp*[tiab] OR referral[tiab] OR “Mass Screening”[Mesh] OR “Referral and Consultation”[Mesh])) with iterative addition of (“access to care” OR “common risk factor” OR “competency-based education” OR “special care dentistry” OR realist).

Embase (Elsevier; searched 20 Jan 2025; updated 8 Aug 2025)

('child'/exp OR 'adolescent'/exp OR child*:ab,ti) AND ('tooth disease'/exp OR 'oral health'/exp OR caries:ab,ti OR dental:ab,ti) AND (outreach:ab,ti OR 'mass screening'/exp OR camp*:ab,ti OR referral:ab,ti) AND [english]/lim, with iterative theory-block terms as above.

Cochrane Library (CENTRAL + Reviews; searched 22 Jan 2025)

MeSH descriptor [Dental Health Services] and [Mass Screening] explode all trees, combined with (child* OR school*):ti,ab,kw AND (dental OR oral):ti,ab,kw; the school dental screening review [ref 4] was identified here.

Web of Science Core Collection (searched 25 Jan 2025; updated 9 Aug 2025)
TS=((child* OR adolescen* OR school*) AND (dental OR "oral health" OR caries) AND (outreach OR screening OR camp* OR referral)) refined to English articles and reviews; theory-block terms added iteratively.

CINAHL (EBSCO; searched 28 Jan 2025)
(MH "Child+" OR MH "Adolescence+") AND (MH "Oral Health+" OR MH "Dental Caries+") AND (MH "Health Screening+" OR outreach OR camp* OR referral); used particularly for nursing, carer-mediation and special-care literature.

Google Scholar (scoping and citation tracking; Feb–Aug 2025)
Purposive scoping and forward/backward citation tracking of key nodes (Pawson & Tilley; Penchansky & Thomas; Sheiham & Watt; Singh et al.; the Cochrane review; WHO reports). Used to locate policy/grey literature and to confirm saturation; first ~10 pages screened per query.

Policy and grey literature (hand-searched; ongoing to Aug 2025)
WHO resolutions and reports (WHA74.5; WHA75.11; WHA76.9; 2022 Status Report; 2024 consolidated Strategy and Action Plan; 2024 Bangkok Declaration), the Lancet 2019 oral health series, and Government of India / NITI Aayog sources (Ayushman Bharat; Viksit Bharat @2047).

Stopping rule. Iterative searching and citation tracking continued until theoretical saturation, operationally defined as two consecutive rounds returning no document that added a new configuration, a new contextual modifier, or a material qualification to an existing mechanism. Saturation was judged to be reached at the 22 August 2025 verification pass.

Supplementary Materials S2. Inclusion/Exclusion Criteria and Document-Selection Flow

S2.1. Eligibility criteria

Criterion	Included	Excluded
Population	Predominantly children/adolescents, aged 0–18 years; mixed child/adult populations where child-relevant findings could be separately discerned	Adults only with no extractable child-relevant inference
Topic	Oral health outreach (school screening, community/anganwadi camps, mobile units, charitable events for CSHCN, satellite outpatient services) and the educational, workforce, financing and policy conditions bearing on these	Clinical/laboratory studies of dental materials or techniques with no public-health outreach dimension
Contribution to theory	Any inference about contexts, mechanisms or outcomes through which outreach did or did not convert into care	Purely descriptive prevalence/epidemiological reports with no contextual or mechanistic content (used only for burden framing)
Document type	Quantitative, qualitative, mixed-methods, conceptual/methodological articles; policy and programme reports (appraised for authority, relevance, traceability)	— (no type excluded a priori; appraised on relevance and rigour)
Language/availability	English-language full text available	Full text unavailable or not in English

Appraisal. Documents were appraised on *relevance* (contribution to programme theory) and *rigour* (credibility of the inference drawn), rather than on a fixed methodological hierarchy, per RAMESES [16]. Policy and grey literature were used to characterise the governing context and were explicitly labelled as such rather than treated as empirical evidence of mechanism.

S2.2. Document-selection flow diagram

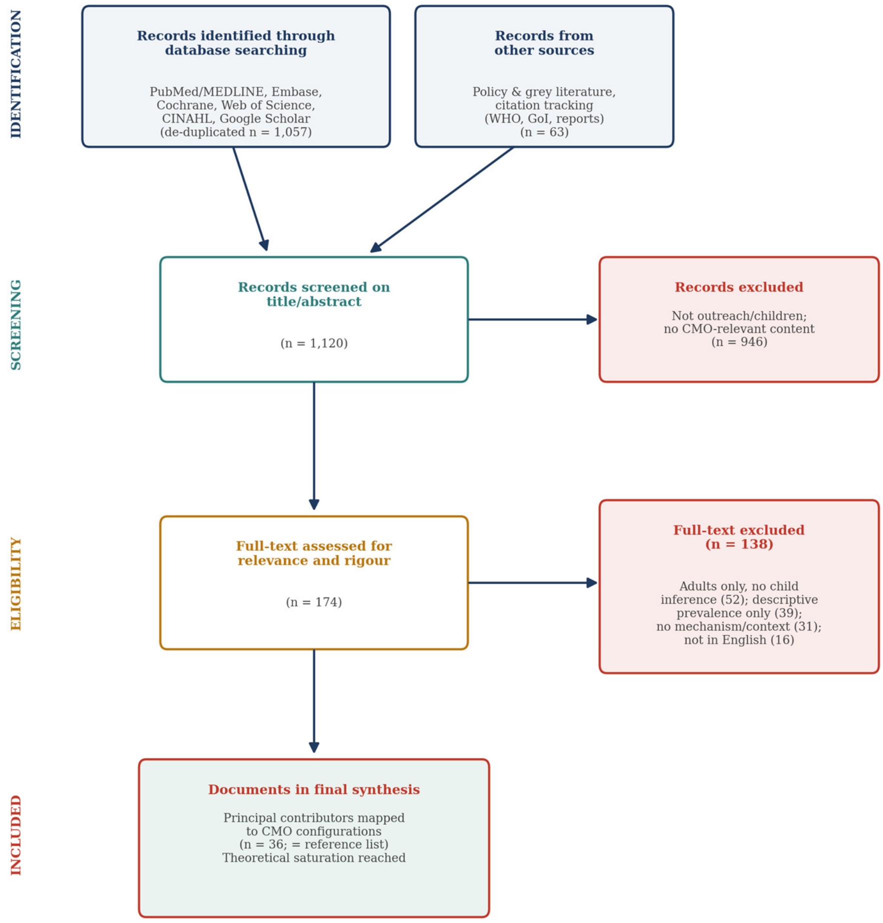

Figure S2-1. RAMESES-consistent document-selection flow. Counts document the purposive, iterative realist process; citation tracking continued until theoretical saturation. Background records consulted during scoping but not cited are not shown as contributors.

Figure S2-1. RAMESES-consistent document-selection flow. Counts document the purposive, iterative realist process; citation tracking continued until theoretical saturation. Background records consulted during scoping but not cited are not shown as contributors to programme theory.

Supplementary Materials S3. Evidence-to-CMO Mapping and RAMESES Checklist

S3.1. Evidence-to-CMO mapping table

The table below records which sources principally informed each configuration and modifier, distinguishes the empirical or conceptual basis from the authors' realist interpretation, and states whether the supporting evidence is drawn directly from children's oral-health outreach or transferred from an adjacent population or field. Reference numbers correspond to the main-text reference list.

Configuration / element	Principal sources	Nature of contribution	Directness of evidence
CMO 1: Caregiver-navigated referral	[3], [18], [20]	Empirical (non-attendance drivers) plus access theory; interpretation of navigation as resource	Direct (camp/outreach) plus general access theory
CMO 2: Trust bridge	[22], [23], [24]	Qualitative (Indian landscape) plus social-accountability education tradition	Partly direct (Indian dental context); education evidence transferred
CMO 3: Risk-factor counselling	[19], [25], [26], [27]	Conceptual (common risk factor approach) plus WHO strategy alignment	Conceptual/policy; not outcome-level in children's outreach
CMO 4: CBE workforce (Compass)	[23], [24], [28]	Empirical/scoping evidence on CBE graduate disposition; interpretation as generative lever	Transferred from dental-education literature (graduate outcomes, not child outcomes)
CMO 5: Closed feedback loop	[11], [12], [13], [14] (continuity principle)	Realist evidence on relationship-based continuity; interpretation for outreach design	Transferred from disability, mental-health and aged-care populations
CMO 6: Affirmation	[11], [12], [18], [20]	Carer-mediated realist evidence plus access theory; affirmation proposed as new dimension	Transferred (carer-mediated) plus conceptual (access theory)
CMO 7: Vulnerable-child optics paradox (antagonist)	[3], [4], [11], [13]	Empirical (low completion; very-low-certainty benefit) plus realist continuity; structural-incentive interpretation	Partly direct (completion/screening); continuity evidence transferred; incentive claim interpretive
CMO 8: Detection without destination (antagonist)	[2], [3], [4], [10]	Empirical plus policy (financing/receiving-system void); mechanism interpretation of absent resource	Direct (screening completion) plus policy context
Cross-cutting modifiers	[2], [21], [22], [35]	Workforce maldistribution, financing, mediation, reporting culture, receiving system	Direct (workforce/policy analyses) plus contextual
Policy ascent/SDG web	[27], [29], [30], [31], [32], [33], [34], [36]	Policy documents characterising the governing context	Policy/contextual, not mechanism evidence

S3.2. Completed RAMESES publication-standards checklist

The checklist below follows the RAMESES publication standards for realist syntheses [16]. “Section” refers to the location in the main manuscript where each item is addressed.

RAMESES item	Description	Addressed in
1. Title	Identifies the report as a realist synthesis	Title; “Realist Synthesis” stated
2. Abstract	Structured summary incl. objective, methods, results, conclusions	Abstract
3. Rationale for review	Explains why the review question suits a realist approach	§1.1
4. Objectives / questions	States what works, for whom, in what contexts, how	§1.1, 1.3
5. Changes in the review process	Documents scope changes during conduct	§2.2
6. Rationale for using realist synthesis	Justifies realist over other methods	§2.1
7. Scope of the review	Defines boundaries and applicability	§1.1 (Scope), 2.2
8. Searching processes	Describes phases, databases, dates, strings, stopping rule	§2.5; Suppl. S1
9. Selection & appraisal of documents	Relevance and rigour criteria; inclusion/exclusion	§2.3, 2.6; Suppl. S2
10. Data extraction	What was extracted and how tagged to configurations	§2.6; Suppl. S3
11. Analysis & synthesis processes	Juxtaposition, reconciliation, adjudication, consolidation	§2.6
12. Document flow diagram	Numbers identified, screened, excluded, included	Suppl. S2 (Fig. S2-1)
13. Document characteristics	Types and provenance of included documents	§3.1; Suppl. S3
14. Main findings (CMOs)	The eight CMO configurations with resource+reasoning	§3.2
15. Programme theory	Initial and refined programme theory	§2.4, 3.3
16. Summary of findings	Refined theory and Constellation of Care	§3.3, 3.4
17. Comparison with literature	Locates findings against prior work	§4.8
18. Strengths, limitations, future research	Reflexivity; testability; prospective evaluation	§4.7, 4.9
19. Conclusions / recommendations	Cautious, testable conclusions; implications by level	§4.10, 5
20. Funding & conflicts	Declared	Back matter

Abbreviations: CMO, Context–Mechanism–Outcome configuration; CSHCN, children with special healthcare needs; RAMESES, Realist And Meta-narrative Evidence Syntheses: Evolving Standards.
